# Electrical Properties of Taste Sensors with Positively Charged Lipid Membranes Composed of Amines and Ammonium Salts

**DOI:** 10.3390/s23198145

**Published:** 2023-09-28

**Authors:** Kentaro Watanabe, Tatsukichi Watanabe, Shunsuke Kimura, Hidekazu Ikezaki, Kiyoshi Toko

**Affiliations:** 1 Graduate School of Information Science and Electrical Engineering, Kyushu University, 744 Motooka, Nishi-ku, Fukuoka 819-0395, Japan; 2 Research and Development Center for Five-Sense Devices, Kyushu University, 744 Motooka, Nishi-ku, Fukuoka 819-0395, Japan; 3 Intelligent Sensor Technology, Inc., 5-1-1 Onna, Atsugi-shi 243-0032, Japan; ikezaki.hidekazu@insent.co.jp; 4 Institute for Advanced Study, Kyushu University, 744 Motooka, Nishi-ku, Fukuoka 819-0395, Japan

**Keywords:** taste sensor, lipid polymer membrane, anion selectivity, Hofmeister series

## Abstract

Currently, taste sensors utilizing lipid polymer membranes are utilized to assess the taste of food products quantitatively. During this process, it is crucial to identify and quantify basic tastes, e.g., sourness and sweetness, while ensuring that there is no response to tasteless substances. For instance, suppression of responses to anions, like tasteless NO_3_^−^ ions contained in vegetables, is essential. However, systematic electrochemical investigations have not been made to achieve this goal. In this study, we fabricated three positively charged lipid polymer membranes containing oleylamine (OAm), trioctylemethylammonium chloride (TOMACl), or tetradodecylammonium bromide (TDAB) as lipids, and sensors that consist of these membranes to investigate the potential change characteristics of these sensors in solutions containing different anions (F^−^, Cl^−^, Br^−^, NO_3_^−^, I^−^). The ability of each anion solution to reduce the positive charge on membranes and shift the membrane potential in the negative direction was in the following order: I*^−^* > NO_3_*^−^* > Br*^−^* > Cl*^−^* > F*^−^*. This order well reflected the order of size of the hydrated ions, related to their hydration energy. Additionally, the OAm sensor displayed low ion selectivity, whereas the TOMACl and TDAB sensors showed high ion selectivity related to the OAm sensor. Such features in ion selectivity are suggested to be due to the variation in positive charge with the pH of the environment and packing density of the OAm molecule in the case of the OAm sensor and due to the strong and constant positive charge created by complete ionization of lipids in the case of TOMACl and TDAB sensors. Furthermore, it was revealed that the ion selectivity varies by changing the lipid concentration in each membrane. These results contribute to developing sensor membranes that respond to different anion species selectively and creating taste sensors capable of suppressing responses to tasteless anions.

## 1. Introduction

Electronic tongues or taste sensors for evaluating human taste perception, one type of chemical sensor, are developed and used as a practical application. The taste sensor developed in Japan is equipped with a lipid polymer membrane on sensor electrodes, allowing the quantification of taste quality and intensity by detecting the voltage between the reference electrode and the sensor electrode [1,2,3,4,5]. The taste sensor imitates human taste perception; each kind of membrane responds to substances classified in each taste quality. This property is called “global selectivity”; taste sensors can identify and quantify the five basic tastes: sourness, saltiness, sweetness, bitterness, and umami. The TS-5000Z taste-sensing system was created and commercialized by Intelligent Sensor Technology, Inc. Over 600 of these taste-sensing systems have been employed globally. Taste sensors are currently used to evaluate various food and beverage items, including coffee, green tea, black tea, ginseng tea, milk, soup, soy sauce, beef, fish, crab, grain, wine, beer, sake, water, juice, amino acids, and dipeptides. Apart from food and drinks, bitterness sensors are employed in research and product development to measure the bitterness of pharmaceuticals. The sensing of taste substances is based on electrostatic interactions and hydrophobic interactions between lipid polymer membranes and taste substances Each taste substance, such as sourness, and saltiness, exhibit different charge states and hydrophobicity/hydrophilicity for each taste quality. The charge state and hydrophobicity/hydrophilicity of a membrane are controlled by adjusting the types and quantities of lipids and plasticizers constituting the lipid polymer membrane, enabling selective quantification of taste qualities [4,5].

Phospholipids are primarily used as negatively charged lipid materials, but their charge state fluctuates with the pH of the solution and interferes with taste measurement in some situations. Our recent study [6,7,8,9] introduced different materials into lipid/polymer membranes as lipids: phosphoric acid di-n-decyl ester (PADE), which is phosphoric acid partially ionized in solution, and tetrakis [3,5-bis(trifluoromethyl)phenyl] borate, sodium salt (TFPB), which is a fully ionized salt. We compared their electrical properties in aqueous solutions containing various kinds of cations. The PADE sensor consistently showed low cation selectivity regardless of lipid concentration, while the TFPB membranes showed high cation selectivity with an increase in lipid concentration [8]. The high cation detection sensitivity by the TFPB sensor reflected the Hofmeister series. It suggests that ion hydration influences the electrostatic interactions with lipid molecules. Furthermore, the disparity in the dissociation states of these two membranes was also reflected in the dependence of the membrane resistance on the lipid concentration.

In addition, utilizing the properties of TFPB sensors unveiled through the above study, we aimed the functional improvement of negatively charged lipid polymer membranes for taste sensors. In the measurement of ampholyte samples, such as amino acids, the degree of proton dissociation in PADE molecules is affected by pH, and hence, it leads to unanticipated changes in membrane potential and influences the sensor response [6]. Therefore, developing a salty taste sensor reflecting the saltiness enhancement effect caused by adding amino acids to table salt was difficult. However, Jing et al. developed a sensor using TFPB instead of PADE and achieved a solution to this problem [7]. The deterioration phenomenon observed in the bitter taste sensor membrane using PADE for pharmaceutical applications was also solved by introducing TFPB [9]. 

As mentioned above, systematic investigations were conducted on the electrical properties of negatively charged lipid polymer membranes, and some applied research using them was also conducted. However, positively charged lipids are predominantly used in commercial taste sensors for food. In one example, a sensor (C00 sensor) to measure the bitterness of food is designed using tetradodecylammonium bromide (TDAB), which is highly hydrophobic, with the expectation of hydrophobic interactions with anionic bitter substances. However, this commercialized sensor exhibits excessive response to tasteless NO_3_*^−^* included in komatsuna and spinach, and hence, it is difficult to evaluate bitterness accurately [10]. Developing novel taste sensors to overcome such a disadvantage requires a systematic understanding of positively charged lipid polymer membranes, which has not been researched. In this study, we fabricated three positively charged lipid polymer membranes containing oleylamine (OAm), trioctylemethylammonium chloride (TOMACl), or TDAB as lipids, and sensors that consist of these membranes to investigate the potential change characteristics of these sensors in solutions containing different anions (F*^−^*, Cl*^−^*, Br*^−^*, NO_3_*^−^*, I*^−^*).

## 2. Materials and Methods

### 2.1. Reagents

**Figure 1:**
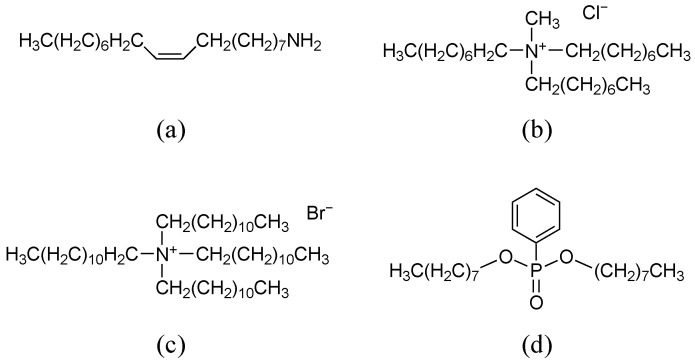
Structure of membrane components: (**a**) OAm; (**b**) TOMACl; (**c**) TDAB; and (**d**) DOPP.

Oleylamine (OAm, Tokyo Chemical Industry Co., Ltd., Tokyo, Japan), methyl tri-n-octylammonium chloride (TOMACl, Tokyo Chemical Industry Co., Ltd., Tokyo, Japan), and tetradodecylammonium bromide (TDAB, Sigma-Aldrich Japan G.K., Tokyo, Japan) were used as positively charged lipids in membranes. Polyvinylchloride (PVC, Fujifilm Wako Pure Chemicals Corporation, Osaka, Japan) was used as the supporting material of membranes. Di-n-octylphenylphosphonate (DOPP, Dojindo Laboratory Co., Kumamoto, Japan) was used as a plasticizer in membranes. Tetrahydrofuran (THF, Sigma-Aldrich Japan G.K., Tokyo, Japan) was used as the organic solvent for forming membranes. Figure 1 shows the structures of the materials comprising three membranes. Potassium fluoride (KF), potassium chloride (KCl), potassium bromide (KBr), and potassium iodide (KI) were purchased from Fujifilm Wako Pure Chemicals Co. (Osaka, Japan). Potassium nitrate (KNO_3_) were purchased from Hayashi Pure Chemical Industry Co. (Osaka, Japan). All reagents were used as received without further purification. 

### 2.2. Preparation of Solutions

Sample solutions were prepared by dissolving KF, KCl, KBr, KNO_3_, and KI in pure water at concentrations of 0.1, 1, 10, 100, and 1000 mM. A standard solution required for determining reference potentials of sensors containing tartaric acid (0.3 mM) and KCl (30 mM) and a cleaning solution containing KCl (100 mM) and KOH (10 mM) in 30% (*v*/*v*) aqueous ethanol were prepared.

### 2.3. Formation of Lipid Polymer Membranes and Fabrication of Sensor Electrodes

**Figure 2:**
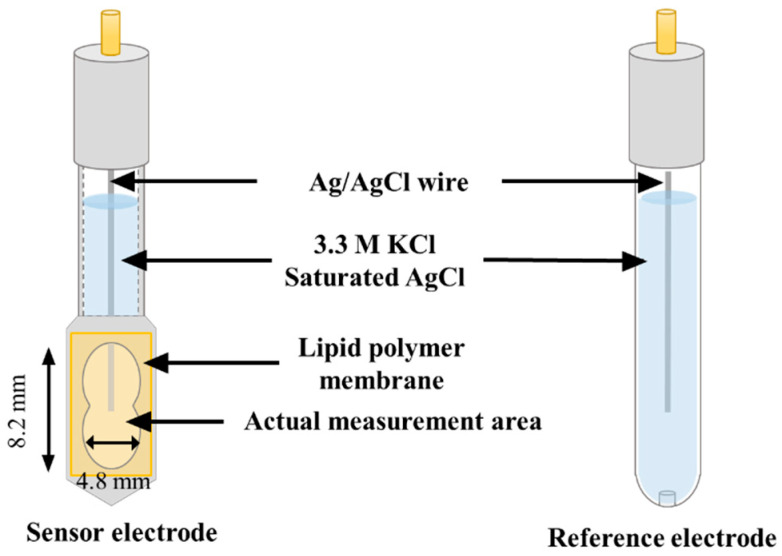
Constructions of taste sensor electrodes.

Lipids (OAm, TOMACl, TDAB), 1 mL of DOOP, and 800 mg of PVC were dissolved in THF. Then, prepared gel solutions were poured into 90 mm Petri dishes to evaporate THF. The thickness of the membranes was ca. 0.35 mm; thus, the volume of membranes was 45 mm × 45 mm × π × 0.35 mm. Therefore, the concentrations of lipids in the membranes prepared in this study were determined as 1, 10, and 100 mM. Completed membranes on Petri dishes were divided into pieces and attached to a hollow sensor probe with an adhesive composing of PVC and THF. Finally, an internal aqueous solution (3.33 M KCl, saturated with AgCl) was injected, and a Ag wire coated with AgCl was inserted. Figure 2 (left) shows the structure of the sensor electrode. Four equal sensors were fabricated from a single Petri dish, i.e., forty-eight sensors (three types of lipids × four concentrations × four electrodes) were fabricated for measurements. For stable potentials, sensors were immersed in the standard solution for 72 h to re-arrange the lipids on the membrane surface before the measurement.

### 2.4. Apparatus

A taste-sensing system (TS-5000Z, Intelligent Sensor Technology, Inc., Kanagawa, Japan) was used to measure the electrochemical potentials of sensors in various solutions automatically. An electrochemical analyzer (ALS 440C, CH Instruments Inc., Austin, TX, USA) was used to analyze the resistance of membranes by voltametric methods.

### 2.5. Measuring Procedure of Electrochemical Potentials of Sensors and Calculation of Relative Values

The measurement procedure was performed in the same way as previously reported [8]. A Ag/AgCl (3.33 M KCl) electrode (Figure 2 (right)) was used as a reference electrode. First, sensors were immersed in a standard solution to record reference potential (*V*_r_), then moved into sample solutions to record a sample potential (*V*_s_). The difference between *V*s and *V*_r_ (*V*_s_ − *V*_r_) was determined as the relative value, i.e., the response value of sensors at various solutions. Finally, sensors were restored to their initial conditions by rinsing with the cleaning solution [11]. The above procedure was repeated in five cycles for each of the four sensors. This study adopted the third to fifth cycle measurement, i.e., the relative values were the average of twelve measurement values (four sensors × three cycles).

### 2.6. Calculation of Selectivity Coefficient

From the changes in relative values against changes in sample concentrations, selectivity coefficients for each sensor for each anion were calculated. The calculation was carried out by the separate solution method (SSM) [12], and a bromide anion was adapted as a reference material. The detailed calculation is shown below.

(1) $$\log K_{i,j}^{pot}=\frac{C_{i}}{C_{j}^{\frac{Z_{i}}{Z_{j}}}}$$

When two solutions are each measured by the sensor, the relative value of solution I at a certain concentration *C_i_* is used as a reference to obtain the concentration *C_j_* when solution J provides the same response, which is calculated from the above equation.

### 2.7. Measurement of Membrane Resistance

The current–potential characteristics of sensor electrodes were measured in the standard solution to calculate the membrane resistance. The potential sweep range was from −100 mV to +100 mV (vs. Ag/AgCl), and the scan rate was 40 mV/s.

## 3. Results and Discussion

### 3.1. Sensor Response and Selectivity Coefficients to Monovalent Anions Using Different Positively Charged Lipid Membranes

**Figure 3:**
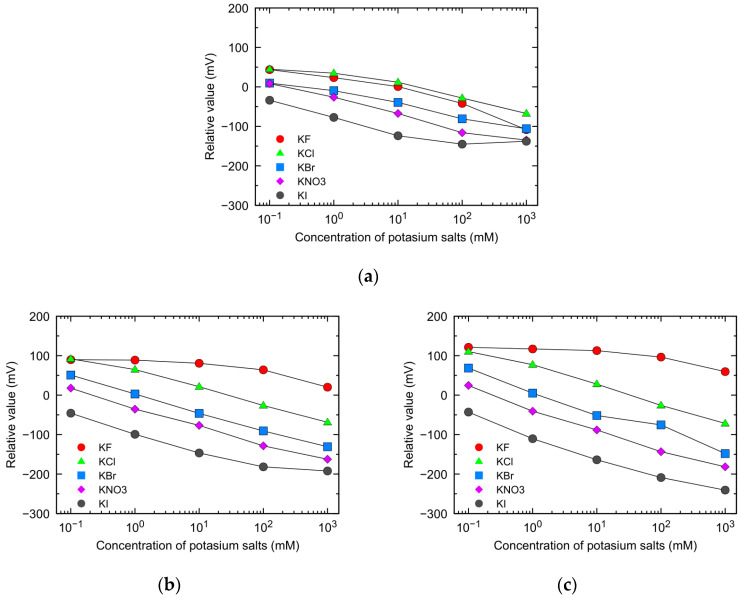
The relative values in various concentrations of monovalent anion solutions using sensors with 10 mM positively charged lipids: (**a**) OAm sensor; (**b**) TOMACl sensor; and (**c**) TDAB sensor.

Figure 3 shows the relative values in potassium salt solutions (0.1 to 1000 mM) measured by sensors equipped with lipid polymer membranes containing 10 mM of OAm, TOMACl, or TDAB. The standard deviation of the relative values was below 15 mV under all measurement conditions. In all sensors and sample solution types, the relative values shifted towards the negative direction as the sample concentration increased. This was because anions mitigated the positive charge caused by cations from lipids in the membrane. Table 1 shows the reference potential (*V*_r_) of the sensors and the selectivity coefficients for various anions (F^−^, Cl^−^, Br^−^, NO_3_^−^, I^−^) in the three types of sensors, as calculated from the measurement results. The selectivity discussed in this study refers to the affinity of the anion for the membrane surface charges. Despite the equal molar quantities of positively charged lipid molecules included in the membrane, *V*_r_ was of the order of TDAB sensor ≅ TOMACl sensor > OAm sensor. This suggests that the charge density of the TDAB sensor and TOMACl sensor is higher than that of the OAm sensor. TOMACl and TDAB molecules, quaternary ammonium salts, ionize completely and acquire a positive charge on their membrane surface. On the other hand, the OAm molecule, which is a primary amine, indicates a partial positive charge due to the repulsion of the same type of charge. It is considered that these differences contribute to the respective *V*_r_ values of each sensor.

**Figure 4:**
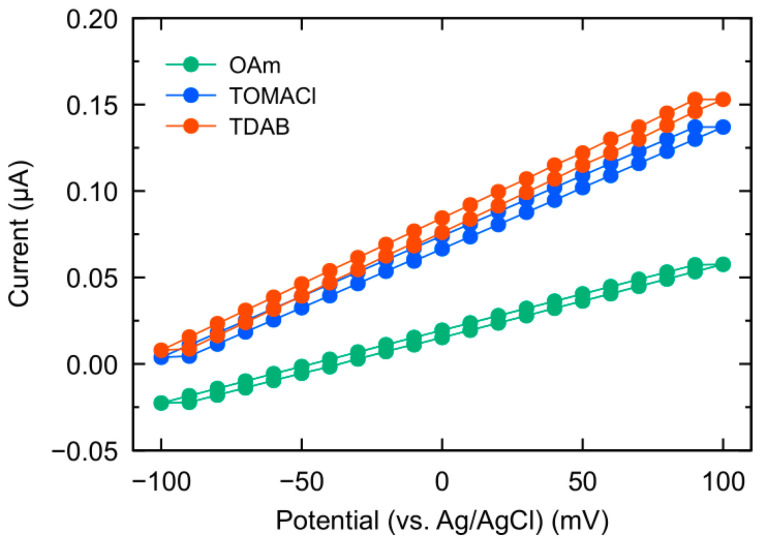
Current–potential characteristics of the three kinds of sensors.

Comparison of membrane resistances in three kinds of membranes can also be helpful in estimating the degree of ionization of lipid molecules. The membrane fabricated in this study supports substances such as amine and ammonium salts ionizing in the PVC/plasticizer matrix. Therefore, the ionization of these substances at the surface and inside the membrane of the PVC/plasticizer matrix structure decreases the membrane resistance due to an increase in anions moving across the membrane, although the situation is very different from that of a complete electrolyte solution. In fact, an increase in charged lipid molecules that constitute the PVC/plasticizer matrix structure also resulted in a decrease in membrane resistance of the negatively charged membrane system [8]. Figure 4 shows the current–potential characteristics of three kinds of sensor. All sensors exhibited a linear relationship in their current–potential characteristics. Still, due to the membranes’ capacitance, their currents did not perfectly match between the case of the positive sweep direction and that of negative direction. The resistance values were 6.9 × 10^6^ Ω·cm^2^ (OAm sensor), 3.6 × 10^6^ Ω·cm^2^ (TOMACl sensor), and 2.9 × 10^6^ Ω·cm^2^ (TDAB sensor). The resistance of the TOMACl and TDAB sensors, which are quaternary ammonium salts, is approximately half of the OAm sensor. This result supports that TOMACl and TDAB molecules become fully ionized and positively charged on the membrane interface.

Based on Figure 3a, the relative value (*V*_s_ − *V*_r_) of the OAm sensor in 30 mM KCl solution is estimated to be −10 mV. This is because the charge state of OAm molecules in the sample solution (30 mM KCl solution) is negative compared to that in the standard solution (30 mM KCl + 0.3 mM tartaric acid) with higher pH. Therefore, *V*_s_ is lower than *V*_r_. On the other hand, based on Figure 3b,c, the relative values of TOMACl sensor and TDAB sensor in 30 mM KCl are estimated to be 0 mV. This was due to the fact TOMACl and TDAB molecules remain ionized regardless of the pH, resulting in no difference in their charge states between the standard solution and 30 mM KCl solution. It implies that there should be no difference in the values of *V*_s_ and *V*_r_, to result in *V*_s_ − *V*_r_ = 0.

Focusing on the variability of relative values resulting from the differences in anion species, the OAm sensor exhibited smaller variation compared to the TOMACl and TDAB sensors in all potassium salt concentration samples. The selectivity coefficients calculated as the Br^−^ ion as the standard (Table 1) help assess this variation. For instance, the difference in selectivity coefficients between F^−^ ion and I^−^ ion for the OAm sensor is 2.94, whereas for the TOMACl sensor and TDAB sensor, it is 9.09 and 12.70, respectively. These results indicate that the selectivity of the OAm sensor towards monovalent anions is significantly lower than that of the TOMACl sensor and TDAB sensor. Furthermore, the selectivity coefficients regarding the TOMACl sensor and TDAB sensor aligned with the Hofmeister series, following the order of I^−^ > NO_3_^−^ > Br^−^ > Cl^−^ > F^−^. In the previous study [8] using sensors composed of fully ionized negatively charged lipids (TFPB), the order of magnitude of cation selectivity coefficients coincided with the Hofmeister series. The results in this study can be explained by a similar mechanism. Many liquid membrane-type ion electrodes and lipid polymer membranes exhibit similar ion selectivity, and their selectivity coefficients follow the Hofmeister series [13,14,15,16,17]. This is attributed to the relationship between the Hofmeister series and the magnitude of ion hydration energy, which is influenced by the ion radius. For instance, ions with high hydration energy, such as F^−^ ions, exhibit weak electrostatic interaction with the membrane interface, resulting in smaller selectivity coefficients. In contrast, ions with low hydration energy, like I^−^ ions, exhibit strong electrostatic interactions with the membrane interface, leading to increased selectivity coefficients.

Particularly, the small selectivity coefficients of TOMACl sensor and TDAB sensor towards F^−^ ions were significant in this study. These sensors showed little shift variation in relative values with increasing F^−^ ion concentrations. This can be attributed to the notably strong hydration of F^−^ ions, making it difficult for the positive charges of lipids to be shielded even in higher concentrations of F^−^ ions. For instance, the relative value of 1000 mM KF measured by the TDAB sensor is 59.5 mV. This significant plus value indicates a weaker shielding of positive charges by 1000 mM F^−^ ions compared to the shielding by Cl^−^ ions in the standard solution composed of 30 mM KCl.

Furthermore, the relative value shift of the OAm sensor with increasing F^−^ ion concentration was remarkable. It shows similarities with the changes observed in Br^−^ and Cl^−^ ion solutions. A selectivity coefficient (−0.61) was much larger than the selectivity coefficients of the TOMACl sensor and TDAB sensor for F^−^ ions. The significant response of the OAm sensor to F^−^ ions can be attributed to two main reasons. First is the pH dependence of the charge state of OAm molecules; some ionized amino groups responsible for the positive charge in the standard solution deprotonated in the weakly basic KF aqueous solution, leading to a loss of positive charge. Thus, it is expected that as the concentration of KF in the aqueous solution increases, the relative value will shift toward the negative direction. The second reason is a strong abstraction effect from the ionized OAm molecules attributed to the extremely high electronegativity of the F^−^ ion [18]. Proton in the ionized amino group of OAm responsible for the positive charge is expected to form a bond with the free F^−^ ions present in the sample solution, shifting the relative value towards the negative direction. The mechanism of this change in relative value differs from the response mechanism observed when relatively weakly hydrated anions (Cl^−^, Br^−^, NO_3_^−^, I^−^) strongly interact with the ionized OAm sensor membrane interface through electrostatic interactions. The interaction between weakly hydrated anions and the sensor membrane interface through electrostatic interactions is particularly clear when the membrane has a high charge density (TOMACl and TDAB sensors), resulting in significant Nernst responses, as shown in Figure 3b,c.

### 3.2. Changes in Monovalent Anion Selectivity with Different Lipid Concentrations

Next, sensors were prepared from membranes containing lipid at different concentrations, and the influence of lipid concentration on the electrical characteristics of the sensors was investigated.

**Figure 5:**
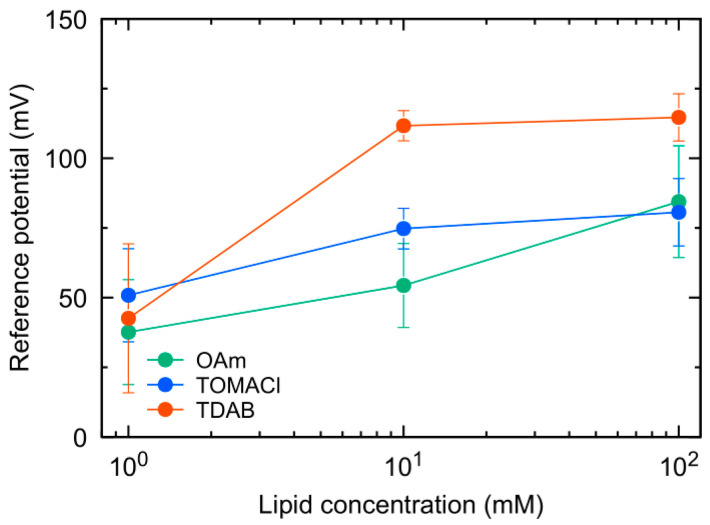
Change in reference potential (*V*_r_) of three kinds of sensor with the lipid concentration.

Figure 5 shows the *V*_r_ of the nine prepared sensors. In the case of the OAm sensor, *V*_r_ shifted monotonically in the positive direction with an increase in lipid content. Similarly, both the TOMACl sensor and the TDAB sensor showed significant positive shifts in *V*_r_ when the lipid content increased from 1 mM to 10 mM. However, no significant difference was observed between 10 mM and 100 mM lipid concentrations. TOMACl and TDAB molecules ionize entirely in aqueous solutions regardless of the pH. However, the membrane potential was considered to saturate at high charge densities, such as a lipid content of 100 mM [13,19]. Moreover, an oil film on the membrane surface was observed in the case of the TDAB sensor with a lipid content of 100 mM. It suggests an inadequate fixation of TDAB molecules to the membrane, which may have interfered with the positive shift in membrane potential.

**Figure 6:**
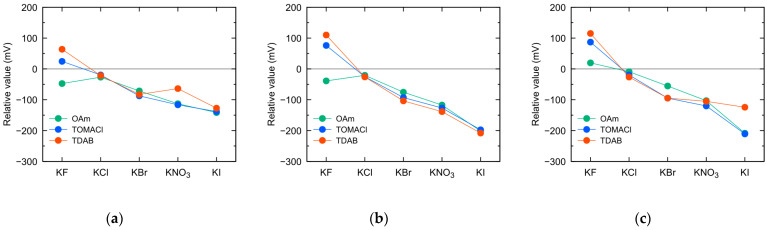
Relative values of sensors using membranes containing lipids (OAm, TOMACl, TDAB) at (**a**) 1 mM; (**b**) 10 mM; and (**c**) 100 mM concentration in 100 mM potassium salt solution.

Figure 6 shows the relative values recorded by sensors with membranes containing lipids (OAm, TOMACl, TDAB) at concentrations of 1 mM, 10 mM, and 100 mM in 100 mM potassium salt (KF, KCl, KBr, KNO_3_, KI)-containing aqueous solutions. An increase in lipid content resulted in a negative shift in the relative values for Cl^−^, Br^−^, NO_3_^−^, and I^−^ ions in all the sensors using different lipids. This can be attributed to an increase in the positive charge at the sensor membrane interface, which corresponds to an increase in the anion binding sites, leading to an improved sensitivity to these anions. However, the negative shift in relative values between sensors with 10 mM and 100 mM lipid content was marginal, due to the saturation of the potential response caused by the strong positive charge of the membrane [13,19,20]. When measuring I^−^ ions using the TDAB sensor, a distinctive trend was indicated where the relative values shifted in the positive direction as the lipid content increased from 10 mM to 100 mM (Figure 5b,c). This unexpected trend matches the characteristics of previously studied negatively charged lipid polymer membrane sensors composed of high concentration of TFPB [8]. Insufficiently fixed TDAB molecules at the membrane interface are considered to contribute to this phenomenon. On the other hand, with an increase in the lipid content, the relative values for F^−^ ions shifted in the positive direction. This can be attributed to the fact that when the sensor has a higher lipid content and a stronger positive charge at the membrane interface, it is less shielded by F^−^ ions that are strongly hydrated. For instance, the differences in relative values for each anion species were made clear when the lipid content was increased. These results indicate that increasing the lipid content is effective in improving anion selectivity.

## 4. Conclusions

In this study, we fabricated three positively charged sensor membranes, which have different charged states, containing OAm, TOMACl, and TDAB. We investigated the potential changes of these sensors in solutions containing various anion species. The ion selectivity of the OAm sensor was significantly lower than the other sensors. It was due to the difference between OAm, whose charge varies depending on the environment, and ammonium salts, which are fully ionized and positively charged at all times. Furthermore, the magnitude of selectivity coefficients of TOMACl and TDAB sensors was I^−^ > NO_3_^−^ > Br^−^ > Cl^−^ > F^−^. The order matched the Hofmeister series and is related to the hydration energy of ions due to their ionic radius. This result suggests that the hydration of anions in the sample solution significantly influences the electrostatic interaction of anions and membrane interface.

Additionally, increasing the lipid content in a membrane resulted in improved ion selectivity. Still, the effect of excessive positive charge of a membrane on its potential response was limited.

The obtained results in this study show, e.g., the possibility of identifying NO_3_^−^ and I^−^, which interfere with measurement, by combining several sensors, and contribute to the design of novel taste sensors. We are researching and developing a novel food bitterness taste sensor that is not sensitive to NO_3_^−^ ions and I^−^ ions. The results will be published soon. Furthermore, research on taste sensors is currently in progress to improve the detection of sweet substances, which are non-charged substances, and umami substances, which show a synergistic effect between monosodium glutamate (MSG) and disodium inosinate (IMP). It is also expected that these issues can be solved by adjusting the charge state of the sensor membrane surface according to the hydration state of the measuring sample.

## Figures and Tables

**Table 1:** Reference potential (*V*_r_), and selectivity coefficients of the sensors.

		Membrane Component		
		OAm (10 mM)	TOMACl (10 mM)	TDAB (10 mM)
*V*_r_ (mV)		48.0	74.5	115.8
Selectivity Coefficients $(\log k_{Br,j}^{pot}$)	F^−^	−0.61	−7.01	−10.64
	Cl^−^	−1.34	−1.52	−1.61
	Br^−^	0	0	0
	NO_3_^−^	0.74	0.68	0.66
	I^−^	2.33	2.08	2.06

## Data Availability

The data presented in this study are available on request.
